# Population Attributable Fraction of Mortality Associated with Tobacco Smoking in Japan: A Pooled Analysis of Three Large-scale Cohort Studies

**DOI:** 10.2188/jea.JE2007429

**Published:** 2008-12-17

**Authors:** Kota Katanoda, Tomomi Marugame, Kumiko Saika, Hiroshi Satoh, Kazuo Tajima, Takaichiro Suzuki, Akiko Tamakoshi, Shoichiro Tsugane, Tomotaka Sobue

**Affiliations:** 1 Cancer Information Services and Surveillance Division, Center for Cancer Control and Information Services, National Cancer Center, Tokyo, Japan; 2 Environmental Health Sciences, Tohoku University Graduate School of Medicine, Sendai, Japan; 3 Aichi Cancer Center Research Institute, Nagoya, Japan; 4 Department of Cancer Control and Statistics, Osaka Medical Center for Cancer and Cardiovascular Diseases, Osaka, Japan; 5 Department of Public Health, Aichi Medical University School of Medicine, Nagakute, Aichi, Japan; 6 Epidemiology and Prevention Division, Research Center for Cancer Prevention and Screening, National Cancer Center, Tokyo, Japan

**Keywords:** Cohort Studies, Population, Risk, Smoking

## Abstract

**Background:**

Quantitative measures of the burden of tobacco smoking in Asian countries are limited. We estimated the population attributable fraction (PAF) of mortality associated with smoking in Japan, using pooled data from three large-scale cohort studies.

**Methods:**

In total, 296,836 participants (140,026 males and 156,810 females) aged 40-79 years underwent baseline surveys during the 1980s and early 1990s. The average follow-up period was 9.6 years. PAFs for all-cause mortality and individual tobacco-related diseases were estimated from smoking prevalence and relative risks.

**Results:**

The prevalence of current and former smokers was 54.4% and 25.1% for males, and 8.1% and 2.4% for females. The PAF of all-cause mortality was 27.8% [95% confidence interval (CI): 25.2-30.4] for males and 6.7% (95% CI: 5.9-7.5) for females. The PAF of all-cause mortality calculated by summing the disease-specific PAFs was 19.1% (95% CI: 16.0-22.2) for males and 3.6% (95% CI: 3.0-4.2) for females. The estimated number of deaths attributable to smoking in Japan in 2005 was 163,000 for males and 33,000 for females based on the former set of PAFs, and 112,000 for males and 19,000 for females based on the latter set. The leading causes of smoking-attributable deaths were cancer (61% for males and 31% for females), ischemic heart diseases and stroke (23% for males and 51% for females), and chronic obstructive pulmonary diseases and pneumonia (11% for males and 13% for females).

**Conclusion:**

The health burden due to smoking remains heavy among Japanese males. Considering the high prevalence of male current smokers and increasing prevalence of young female current smokers, effective tobacco controls and quantitative assessments of the health burden of smoking need to be continuously implemented in Japan.

## INTRODUCTION

Smoking is a major preventable cause of premature mortality. Estimating the mortality attributable to smoking is necessary in order to assess the health burden that it causes within a population, and such estimates have accordingly been performed in many countries and regions.^1^^-^^5^ In Japan, recent studies have estimated the population impact of smoking on selected causes of death, including all causes,^6^ all cancers,^7^ lung cancer,^8^ pancreatic cancer,^9^ and cardiovascular diseases.^10^ Since smoking causes many diseases, including numerous other types of cancer and cardiovascular, respiratory, and digestive diseases,^11^^,^^12^ a comprehensive approach is needed to fully understand its health burden. Single cohort studies, however, do not include sufficiently large sample sizes to enable examination of the health effects of smoking on diseases with low mortality or incidence rates, particularly among populations with a low prevalence of smoking such as Japanese females. A historical large-scale cohort study in Japan, the Hirayama study, estimated the fraction of deaths attributable to smoking for many diseases among approximately 265,000 participants.^13^ The baseline survey for the Hirayama study was conducted in 1965, and the follow-up was continued until the end of 1982. In the nearly 40 years since the Hirayama study began, the list of diseases known to be caused by smoking has been altered and expanded.^12^ The purpose of the present study was, therefore, to estimate the population attributable fraction (PAF) of mortality caused by smoking in Japan in a comprehensive manner, based on the updated list of smoking-related diseases, and using data from nearly 300,000 participants of three large-scale Japanese cohort studies.

## METHODS

### Study Population

The present study used pooled data from three ongoing prospective studies in Japan: (1) the Japan Public Health Center-based Prospective Study (JPHC study),^14^ which comprises two different cohorts (JPHC-I and JPHC-II) with different baseline survey years; (2) the Three-Prefecture Cohort Study (3-pref study);^15^ and (3) the Japan Collaborative Cohort Study (JACC study).^16^^,^^17^ For each cohort, we collected baseline and follow-up data from each of the participants aged 40-79 years at baseline (40-59 years for the JPHC-I cohort, 40-69 years for the JPHC-II cohort, and 40-79 years for the 3-pref and JACC cohorts). The numbers of participants in the original dataset collected from each cohort were 61,595 for the JPHC-I cohort, 78,825 for the JPHC-II cohort, 108,774 for the 3-pref cohort, and 110,792 for the JACC cohort. For participant selection, we applied the following exclusion criteria: (1) moving out of the study area before the beginning of the follow-up, (2) ineligible age (younger than 40 years or older than 80 years), and (3) unknown outcome. We applied the following additional exclusion criteria to the data from the JPHC-I and JPHC-II cohorts: (1) foreign nationality, (2) refusal to participate in the follow-up, (3) duplicate registration, and (4) unavailability of baseline questionnaire data. The number of participants in each cohort after the exclusion criteria had been applied was 50,217 for the JPHC-I, 63,189 for the JPHC-II, 104,876 for the 3-pref, and 110,792 for the JACC. From the combined 329,074 (148,929 males and 180,145 females) participants, we excluded 4,283 (1,719 males and 2,564 females) duplicates who were enrolled in both the 3-pref study and the JACC study, and 27,955 (7,184 males and 20,771 females) participants who had incomplete smoking data. As a result, 296,836 participants (140,026 males and 156,810 females) were included in the analysis, which covered 26 of Japan’s 47 prefectures (55%). The characteristics of the participants included in the analysis are summarized in Table 1. This pooled study was approved by the institutional review board of the National Cancer Center, Japan.

**Table 1.** Characteristics of the pooled cohort studies and participants

Cohort	Area	Participants characteristics	Baseline year	End of follow-up	Average follow-up years (SD)	Sex	n	Age at baseline (year)		Smoking status at baseline (%)		
	
								Average (SD)	Range	Current	Former	Never
JPHC-I	5 public health center areas in Iwate, Akita, Nagano, Okinawa, and Tokyo prefectures	Residents in each public health center area in the first 4 prefectures; participants of a health checkup in Tokyo Prefecture	1990	December 31, 2000	10.4 (1.6)	Male	23,478	49.0 (6.0)	40-59	12,589 (53.6%)	5,428 (23.1%)	5,461 (23.3%)
			(One area^†^; 1990-1994)			Female	26,561	49.1 (5.9)	40-59	2,090 (7.9%)	656 (2.5%)	23,815 (89.7%)

JPHC-II	6 public health center areas in Ibaraki, Niigata, Kochi, Nagasaki, Okinawa, and Osaka prefectures	Residents in each public health center area in the first 5 prefectures; participants of a health checkup in Osaka Prefecture	1993-1994	December 31, 2003	10.2 (1.7)	Male	29,567	53.2 (8.8)	40-69	15,383 (52.0%)	7,246 (24.5%)	6,938 (23.5%)
						Female	33,175	53.5 (8.9)	40-69	2,435 (7.3%)	502 (1.5%)	30,238 (91.1%)

3-pref	10 cities, towns, or wards in Miyagi, Aichi, and Osaka prefectures	Residents in each area	Feb. 1, 1983-Nov 1, 1985	Jan. 31, 1993-Oct. 31, 1995	8.5 (2.7)	Male	44,453	54.4 (10.2)	40-79	25,699 (57.8%)	11,164 (25.1%)	7,590 (17.1%)
			(One area^‡^ Dec. 1, 1990)	(One area^‡^ Feb. 28, 2000)		Female	43,704	55.2 (10.5)	40-79	5,188 (11.9%)	1,631 (3.7%)	36,885 (84.4%)

JACC	45 cities, towns, or villages in 18 prefectures* throughout Japan, except Shikoku district	Residents in 22 areas; participants of a health checkup in 20 areas; combination of these two or atomic bomb survivors in the remaining 3 areas	1988-1990	December 31, 1999	9.9 (2.2)	Male	42,528	57.3 (10.2)	40-79	22,556 (53.0%)	11,241 (26.4%)	8,731 (20.5%)
						Female	53,370	57.3 (10.1)	40-79	3,004 (5.6%)	925 (1.7%)	49,441 (92.6%)

Pooled					9.6 (2.3)	Male	140,026	54.1 (9.7)	40-79^§^	76,227 (54.4%)	35,079 (25.1%)	28,720 (20.5%)
						Female	156,810	54.5 (9.8)	40-79^§^	12,717 (8.1%)	3,714 (2.4%)	140,379 (89.5%)

JPHC: Japan Public Health Center-based prospective study, 3-pref: Three-prefecture cohort study, JACC: Japan Collaborative Cohort Study

*: Hokkaido, Akita, Ibaraki, Tochigi, Chiba, Kanagawa, Niigata, Yamanashi, Nagano, Gifu, Shiga, Kyoto, Hyogo, Wakayama, Tottori, Hiroshima, Fukui, and Saga prefectures

†: Katsushika area in Tokyo Prefecture.

‡^:^ Izumi-otsu in Osaka Prefecture.

§: The age distribution (40-49, 50-59, 60-69, and 70-79 years old) of the pooled data was as follows; 35.9%, 35.3%, 21.3%, and 7.5% for males; 34.2%, 35.5%, 22.3%, and 8.0% for females.

SD: standard deviation

### Smoking Assessment

In each of the three studies, smoking habits were assessed by self-administered questionnaires. Although the style of the questions differed slightly,^18^ all of the studies included questions concerning current smoking status, age at initiation of smoking, average number of cigarettes smoked per day, and age at cessation of smoking for former smokers. The smoking status at baseline was classified into three categories: never-smoker, current smoker, and former smoker. Current smokers included occasional smokers (JPHC-I and 3-pref studies).

### Follow-up

The average follow-up period was 9.6 [standard deviation (SD): 2.3] years (Table 1). Residential status, including survival, date of death, and date of moving out of the study area, was confirmed through the residential registries kept in the municipalities of the study areas. Information on the cause of death was confirmed by vital statistics files obtained with official permission.

### Causes of Death

The endpoint of the present study was defined as death during the observation period. We selected the causes of death from the diseases judged to be “causally related” to active smoking in the Surgeon General’s report of 2004^12^ or the International Agency for Research on Cancer (IARC) Monograph volume 83,^11^ and grouped these into “tobacco-related diseases” (the ICD-9 and ICD-10 codes are listed in the Appendix). We also analyzed all-cause deaths and the following four major disease groups: all cancers, all cardiovascular diseases (CVDs), all respiratory system diseases, and all digestive system diseases.

### Statistical Analysis

The person-years of follow-up were calculated from the date of the baseline questionnaire to whichever of the following events occurred first: the end of the follow-up for each study, the date of death, or the date of moving out of the study area. The hazard ratio (HR) and 95% confidence interval (CI) were used to describe the relative risk for current, former, and ever-smokers compared with never-smokers. The Cox proportional hazards model was used to adjust for age (continuous variable), using the SAS^®^ PHREG procedure (version 8.02, The SAS Institute, USA).

In order to express the impact of tobacco smoking on the study population, the PAF (%) was estimated for all causes and specific causes of death. For each disease group, the PAF was calculated using the following equation:

$$\text{PAF}={\text{P}}_{\text{d}}({\text{HR}}_{\text{a}}-1)/{\text{HR}}_{\text{a}},$$ (1)

where P_d_ is the proportion of exposed among those who died of a given cause of death, and HR_a_ is the age-adjusted HR for that cause of death.^19^ The Greenland formula was used to calculate the 95% CI for the PAF.^20^ For all-cause mortality, the PAF was calculated in two ways. The first was by equation (1) using the HR for all-cause mortality. The second was by calculating the weighted sum of the PAF for each disease as follows:

$${\text{PAF}}_{\text{all-cause}}=\Sigma ({\text{PAF}}_{\text{i}}\times {\text{D}}_{\text{i}})/{\text{D}}_{\text{all}},$$ (2)

where PAF_i_ and D_i_ indicate the PAF and the number of deaths, respectively, for each tobacco-related disease i, and D_all_ indicates the number of all-cause deaths. It should be noted that equation (2) assumes that the PAF for diseases other than tobacco-related diseases is zero. The PAF for “total tobacco-related diseases” was calculated by equation (1) using the HR for overall mortality from tobacco-related diseases.

The annual number of smoking-attributable deaths in Japan was calculated using the vital statistics data of 2005 using two methods: first, by multiplying the sex-specific total number of deaths in Japan by the PAF of ever-smoking for all-cause mortality calculated by equation (1); and, second, by summing the sex-specific number of deaths from each tobacco-related disease in Japan weighted by the corresponding PAF of ever-smoking. Since the number of deaths from abdominal aortic aneurysm was not available in the published data, the number of deaths and the PAF of aortic aneurysm and dissection were used instead.

## RESULTS

The prevalence of current and former smoking at baseline among the pooled participants was 54.4% and 25.1% for males and 8.1% and 2.4% for females, respectively (Table 1).

During the 2,855,396 person-years of follow-up (1,325,004 males and 1,530,392 females) for 296,836 participants, a total of 25,700 deaths (male: 16,282, female: 9,418) were recorded. The numbers of deaths from major causes for males were 6,505 (40.0%) for cancer, 4,306 (26.4%) for CVD, 1,587 (9.7%) for respiratory system diseases, and 596 (3.7%) for digestive system diseases. The numbers of deaths from major causes for females were 3,475 (36.9%) for cancer, 2,904 (30.8%) for CVD, 681 (7.2%) for respiratory system diseases, and 320 (3.4%) for digestive system diseases.

### Age-Adjusted HR According to Smoking Status

Table 2 shows the disease-specific, age-adjusted HRs for males according to smoking status. Current smokers had a nearly 1.5-fold higher age-adjusted rate of mortality from all causes, all CVDs, and all respiratory diseases, and a nearly 2.0-fold higher mortality from total tobacco-related diseases, all cancers, and all digestive diseases compared with never-smokers. Among the tobacco-related cancer sites, the larynx exhibited the highest HR point estimate, followed by the urinary tract (renal pelvis, ureter, and bladder), lung, esophagus, lip/oral cavity/pharynx, liver, pancreas, and stomach. Among CVDs, ischemic heart disease (IHD) had a higher HR than stroke. When divided into stroke subtypes, subarachnoid hemorrhage had the highest HR, followed by intracerebral hemorrhage and cerebral infarction. Abdominal aortic aneurysm had an even higher HR; however, this ratio had a wide CI. Chronic obstructive pulmonary diseases (COPD) and peptic ulcer had HRs of 3.0 or higher.

**Table 2.** Disease-specific, age-adjusted hazard ratio according to smoking status for males

Cause of death	Age-adjusted hazard ratio (vs. never-smokers) (95% confidence interval)^†^		

	Current smokers	Former smokers	Ever-smokers
All-cause	1.63 (1.56-1.70)	1.27 (1.21-1.33)	1.49 (1.43-1.55)

Total tobacco-related diseases	1.85 (1.74-1.97)	1.40 (1.30-1.50)	1.67 (1.57-1.78)

All cancers	1.97 (1.83-2.13)	1.50 (1.38-1.63)	1.79 (1.67-1.93)
Total tobacco-related cancers	2.32 (2.12-2.54)	1.64 (1.49-1.82)	2.06 (1.89-2.26)
Lip, oral cavity, and pharynx*	2.66 (1.48-4.77)	1.89 (1.00-3.58)	2.37 (1.34-4.20)
Esophagus*	3.39 (2.25-5.09)	2.22 (1.43-3.46)	2.96 (1.98-4.42)
Stomach*	1.51 (1.29-1.77)	1.28 (1.08-1.52)	1.42 (1.22-1.66)
Liver*	1.81 (1.49-2.20)	1.63 (1.32-2.01)	1.74 (1.44-2.11)
Pancreas*	1.58 (1.18-2.11)	1.19 (0.86-1.65)	1.43 (1.08-1.90)
Larynx*	5.47 (1.29-23.11)	3.03 (0.65-14.01)	4.50 (1.08-18.72)
Lung*	4.79 (3.88-5.92)	2.41 (1.91-3.03)	3.85 (3.12-4.74)
Kidney, except renal pelvis*	1.57 (0.81-3.06)	1.46 (0.71-3.00)	1.53 (0.81-2.90)
Renal pelvis, ureter, bladder*	5.35 (2.47-11.57)	2.76 (1.21-6.31)	4.30 (2.01-9.23)
Myeloid leukemia*	1.45 (0.74-2.82)	2.13 (1.07-4.25)	1.69 (0.89-3.18)

All cardiovascular diseases	1.52 (1.39-1.65)	1.17 (1.07-1.29)	1.38 (1.27-1.49)
Total tobacco-related cardiovascular diseases	1.51 (1.36-1.68)	1.19 (1.06-1.33)	1.38 (1.25-1.53)
Ischemic heart diseases*	2.18 (1.79-2.66)	1.71 (1.39-2.12)	2.00 (1.65-2.42)
Total stroke*	1.25 (1.10-1.42)	1.00 (0.87-1.14)	1.15 (1.02-1.29)
Subarachnoid hemorrhage	2.33 (1.50-3.64)	1.19 (0.71-2.02)	1.94 (1.25-3.00)
Intracerebral hemorrhage	1.24 (0.98-1.57)	0.91 (0.69-1.19)	1.11 (0.89-1.40)
Cerebral infarction	1.23 (1.02-1.50)	1.02 (0.82-1.26)	1.14 (0.95-1.37)
Aortic aneurysm and dissection	3.89 (2.02-7.49)	2.71 (1.35-5.42)	3.42 (1.80-6.51)
Abdominal aortic aneurysm*	3.89 (1.38-10.99)	1.64 (0.52-5.24)	2.94 (1.05-8.18)

All respiratory diseases	1.41 (1.22-1.62)	1.37 (1.18-1.59)	1.39 (1.22-1.59)
Total toabacco-related respiratory diseases	1.35 (1.15-1.59)	1.25 (1.05-1.48)	1.30 (1.12-1.52)
Pneumonia*	1.17 (0.98-1.39)	1.09 (0.91-1.31)	1.13 (0.96-1.33)
Chronic obstructive pulmonary diseases*	3.09 (1.90-5.03)	2.76 (1.68-4.55)	2.95 (1.84-4.72)

All digestive diseases	2.04 (1.60-2.60)	1.22 (0.92-1.62)	1.74 (1.37-2.21)
Peptic ulcer*	7.13 (1.71-29.78)	1.96 (0.40-9.72)	5.01 (1.21-20.77)

*: Tobacco-related diseases selected from the Surgeon General’s Report of 2004 and IARC Monograph volume 83.

†: Cox proportional hazard model

The excess risks for male former smokers were lower than those for male current smokers. The former smokers had lower HRs than the current smokers for the four major disease groups (cancer, CVD, respiratory, and digestive diseases), and also for the subgroups within each category, except myeloid leukemia.

Table 3 shows the disease-specific, age-adjusted HRs for females according to smoking status. The HRs of the current smokers (vs. never-smokers) were nearly 1.7 for all causes, all cancers, and all respiratory diseases, and nearly 2.0 for total tobacco-related diseases, all CVDs, and all digestive diseases. Among the tobacco-related cancer sites, the lung exhibited the highest HR for current smokers, followed by the cervix uteri, lip/oral cavity/pharynx, esophagus, urinary tract, pancreas, liver, and stomach, of which the lung, cervix uteri, pancreas, and liver were significant. As observed for males, IHD had a higher HR than stroke, and subarachnoid hemorrhage had the highest HR among the stroke subtypes, followed by intracerebral hemorrhage and cerebral infarction. A tendency toward a higher HR for abdominal aortic aneurysm was also observed among females. COPD had the highest HR among respiratory and digestive diseases. For total tobacco-related diseases and all CVDs, the HRs of former smokers were smaller than those of current smokers. The HRs of former smokers (vs. never-smokers) were similar to, or higher than, those of current smokers for many other diseases and all-cause mortality.

**Table 3.** Disease-specific, age-adjusted hazard ratio according to smoking status for females.

Cause of death	Age-adjusted hazard ratio (vs. never-smokers) (95% confidence interval)^†^		

	Current smokers	Former smokers	Ever-smokers
All-cause	1.76 (1.65-1.87)	1.68 (1.52-1.86)	1.73 (1.64-1.83)

Total tobacco-related diseases	2.00 (1.83-2.19)	1.65 (1.42-1.91)	1.90 (1.75-2.06)

All cancers	1.57 (1.41-1.75)	1.57 (1.32-1.87)	1.57 (1.43-1.73)
Total tobacco-related cancers	2.01 (1.76-2.30)	1.70 (1.35-2.14)	1.93 (1.71-2.17)
Lip, oral cavity, and pharynx*	1.97 (0.69-5.65)	1.23 (0.17-9.12)	1.76 (0.68-4.59)
Esophagus*	1.90 (0.74-4.86)	3.59 (1.27-10.16)	2.40 (1.15-5.02)
Stomach*	1.22 (0.90-1.64)	1.47 (0.95-2.27)	1.29 (1.00-1.66)
Liver*	1.73 (1.21-2.48)	1.23 (0.63-2.39)	1.59 (1.15-2.20)
Pancreas*	1.81 (1.28-2.57)	1.96 (1.16-3.30)	1.85 (1.37-2.50)
Larynx*	0.00 – –	0.00 – –	0.00 – –
Lung*	3.88 (3.07-4.90)	2.63 (1.72-4.03)	3.55 (2.86-4.40)
Cervix uteri*	2.32 (1.31-4.10)	1.00 (0.25-4.09)	1.99 (1.16-3.41)
Kidney, except renal pelvis*	0.60 (0.08-4.47)	1.55 (0.21-11.52)	0.86 (0.20-3.69)
Renal pelvis, ureter, bladder*	1.86 (0.84-4.11)	0.00 – –	1.30 (0.59-2.88)
Myeloid leukemia*	0.96 (0.30-3.10)	0.96 (0.13-7.01)	0.96 (0.34-2.68)

All cardiovascular diseases	1.98 (1.78-2.21)	1.60 (1.34-1.91)	1.87 (1.70-2.06)
Total tobacco-related cardiovascular diseases	2.09 (1.83-2.39)	1.66 (1.33-2.07)	1.97 (1.75-2.21)
Ischemic heart diseases*	2.95 (2.33-3.73)	2.48 (1.71-3.60)	2.81 (2.28-3.46)
Total stroke*	1.80 (1.52-2.12)	1.35 (1.01-1.79)	1.66 (1.44-1.93)
Subarachnoid hemorrhage	2.79 (2.06-3.78)	1.05 (0.50-2.24)	2.33 (1.75-3.11)
Intracerebral hemorrhage	1.92 (1.39-2.67)	1.69 (0.99-2.89)	1.86 (1.39-2.48)
Cerebral infarction	1.48 (1.10-2.00)	1.17 (0.72-1.91)	1.39 (1.07-1.80)
Aortic aneurysm and dissection	2.35 (1.16-4.79)	3.16 (1.25-7.95)	2.59 (1.43-4.69)
Abdominal aortic aneurysm*	4.30 (1.16-15.96)	6.51 (1.39-30.39)	4.98 (1.66-14.94)

All respiratory diseases	1.65 (1.29-2.09)	1.27 (0.85-1.89)	1.53 (1.24-1.90)
Total toabacco-related respiratory diseases	1.53 (1.13-2.07)	1.39 (0.88-2.21)	1.49 (1.15-1.93)
Pneumonia*	1.39 (1.00-1.93)	1.40 (0.87-2.26)	1.40 (1.06-1.84)
Chronic obstructive pulmonary diseases*	3.55 (1.53-8.21)	1.16 (0.16-8.54)	2.82 (1.27-6.26)

All digestive diseases	2.13 (1.54-2.94)	2.10 (1.28-3.43)	2.12 (1.60-2.81)
Peptic ulcer*	1.37 (0.32-5.94)	1.50 (0.20-11.31)	1.42 (0.42-4.82)

*: Tobacco-related diseases selected from the Surgeon General’s Report of 2004 and IARC Monograph volume 83.

†: Cox proportional hazard model

### PAF of Disease-specific Mortality Due to Smoking

**Figure 1.**
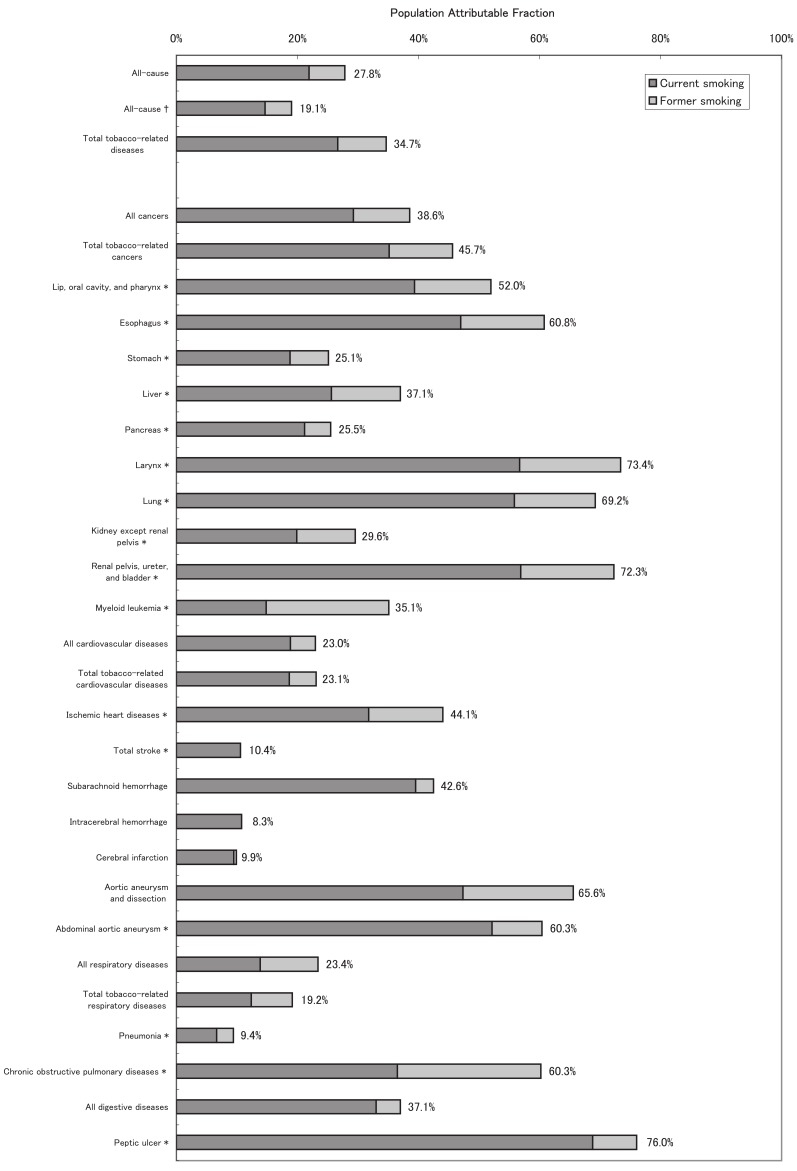
Population attributable fraction of disease-specific mortality due to smoking for males.

Figure 1 shows the male age-adjusted, disease-specific PAFs of current, former, and ever-smoking. After age-adjustment, 28% of the all-cause mortality was attributable to ever-smoking among males. For all cancers, the corresponding PAF was up to 40%. When divided into tobacco-related cancer sites, the larynx, urinary tract, and lung had PAFs of nearly 70%. The PAFs for the esophagus and the lip/oral cavity/pharynx were also greater than 50%, whereas those for the other sites ranged from 25% to 40%. The PAF for all CVDs was approximately 20%, which was smaller than that for all cancers. Among the CVDs, IHD, subarachnoid hemorrhage, and aortic aneurysm had PAFs of over 40%, whereas total stroke and its subtypes other than subarachnoid hemorrhage had PAFs of approximately 10%. The PAFs for all respiratory diseases and all digestive system diseases were approximately 20% and 40%, respectively. COPD and peptic ulcer had PAFs of over 60%.

*: Tobacco-related diseases selected from the Surgeon General’s Report of 2004 and the IARC Monograph volume 83.

†: The population attributable fraction was calculated by summing the attributable fractions estimated for each tobacco-related disease (*).

The percentage shown at the right-hand end of each bar is the population attributable fraction of ever-smoking.

See Appendix for the values of the point estimates and confidence intervals.

**Figure 2.**
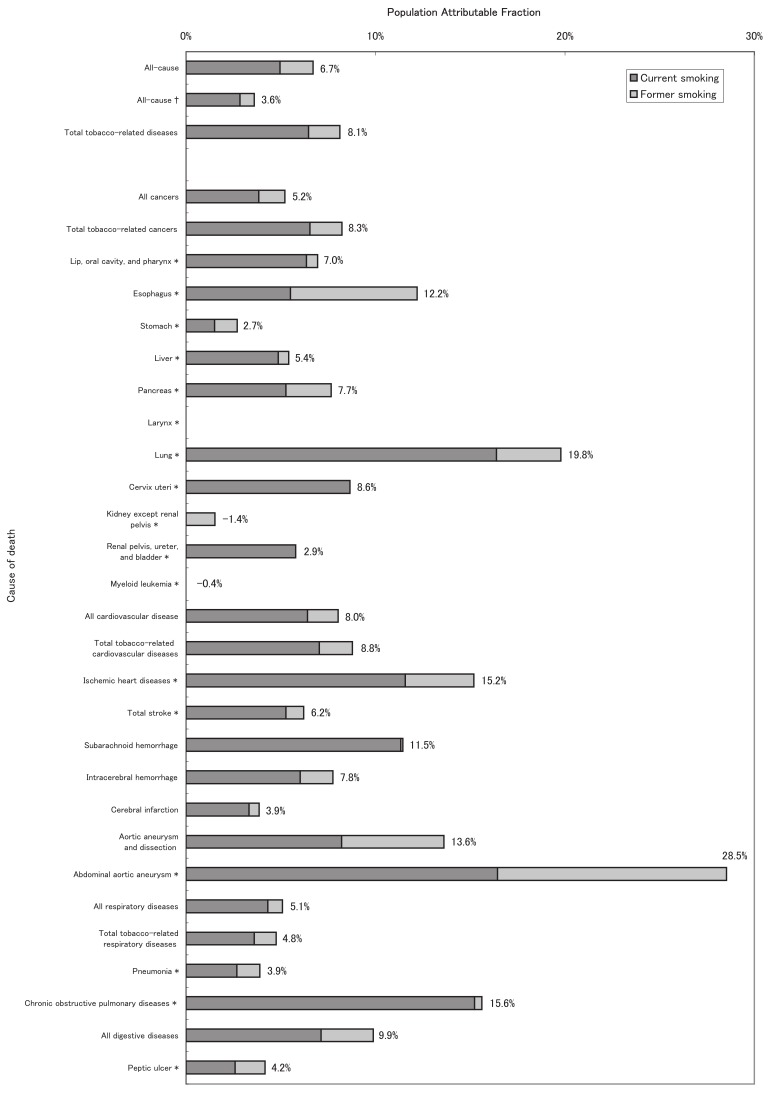
Population attributable fraction of disease-specific mortality due to smoking for females.

Figure 2 shows the female age-adjusted, disease-specific PAFs of current, former, and ever-smoking. After age-adjustment, 7% of the all-cause mortality was attributable to ever-smoking among females, which was a considerably smaller proportion than that for males. For all cancers, the corresponding PAF was also approximately 5%. When divided into tobacco-related cancer sites, the lung had a relatively large PAF (20%), whereas the PAFs for the other sites were approximately 10% or less. The PAF for all CVDs was slightly larger than that for all cancers, but was less than 10%. As was the case among males, IHD, subarachnoid hemorrhage, and aortic aneurysm in females had relatively large PAFs (10-30%). The PAFs for all respiratory diseases and all digestive system diseases were 5% and 10%, respectively. COPD had a relatively large PAF of approximately 15%.

*: Tobacco-related diseases selected from the Surgeon General’s Report of 2004 and the IARC Monograph volume 83.

†: The population attributable fraction was calculated by summing the attributable fractions estimated for each tobacco-related disease (*).

The percentage shown at the right-hand end of each bar is the population attributable fraction of ever-smoking.

See Appendix for the values of the point estimates and confidence intervals.

The PAF of ever-smoking for all-cause mortality, calculated by summing the disease-specific PAFs for tobacco-related diseases, was 19% for males and 4% for females. These values were smaller than those directly calculated from the relative risk of all-cause mortality (28% for males and 7% for females; Appendix).

**Appendix.** Cause-specific, age-adjusted population attributable fraction according to smoking status, for males and females.

Cause of death	ICD-9	ICD-10	Males						Females					
	
			Current smokers		Former smokers		Ever-smokers		Current smokers		Former smokers		Ever-smokers	
All-cause	(All)	(All)	21.9%	(20.1%-23.7%)	5.9%	(4.7%-7.1%)	27.8%	(25.2%-30.4%)	5.0%	(4.3%-5.6%)	1.8%	(1.3%-2.2%)	6.7%	(5.9%-7.5%)
All-cause^§^	(All)	(All)	14.7%	(12.9%-16.4%)	4.4%	(3.5%-5.3%)	19.1%	(16.0%-22.2%)	2.9%	(2.3%-3.4%)	0.8%	(0.5%-1.0%)	3.6%	(3.0%-4.2%)

Total tobacco-related diseases			26.7%	(24.3%-28.9%)	8.0%	(6.4%-9.6%)	34.7%	(31.2%-38.0%)	6.5%	(5.4%-7.5%)	1.7%	(1.0%-2.3%)	8.1%	(6.9%-9.4%)

All cancers	140-208	C00-C97	29.3%	(26.5%-31.9%)	9.3%	(7.5%-11.1%)	38.6%	(34.5%-42.3%)	3.8%	(2.7%-4.9%)	1.4%	(0.7%-2.0%)	5.2%	(3.9%-6.5%)
All cancers^§^	140-208	C00-C97	26.0%	(22.5%-29.5%)	7.8%	(6.2%-9.5%)	33.8%	(27.5%-40.2%)	3.5%	(2.6%-4.5%)	1.0%	(0.5%-1.5%)	4.4%	(3.3%-5.5%)
Total tobacco-related cancers			35.2%	(32.1%-38.1%)	10.5%	(8.5%-12.5%)	45.7%	(41.2%-49.8%)	6.5%	(4.9%-8.2%)	1.7%	(0.8%-2.6%)	8.3%	(6.3%-10.1%)
Lip, oral cavity, and pharynx*	140-149	C00-C14	39.3%	(19.0%-54.6%)	12.7%	(0.3%-23.6%)	52.0%	(19.6%-71.4%)	6.4%	(-7.2%-18.2%)	0.6%	(-6.0%-6.8%)	7.0%	(-8.6%-20.3%)
Esophagus*	150	C15	47.0%	(35.3%-56.5%)	13.8%	(6.7%-20.4%)	60.8%	(43.4%-72.9%)	5.5%	(-5.3%-15.2%)	6.7%	(-2.7%-15.2%)	12.2%	(-2.4%-24.7%)
Stomach*	151	C16	18.8%	(12.1%-24.9%)	6.4%	(2.0%-10.6%)	25.1%	(15.0%-34.1%)	1.5%	(-1.0%-4.0%)	1.2%	(-0.4%-2.8%)	2.7%	(-0.4%-5.7%)
Liver*	155	C22	25.6%	(18.1%-32.5%)	11.5%	(6.7%-16.0%)	37.1%	(25.9%-46.6%)	4.9%	(0.9%-8.7%)	0.6%	(-1.5%-2.6%)	5.4%	(0.9%-9.8%)
Pancreas*	157	C25	21.2%	(8.3%-32.3%)	4.4%	(-3.8%-11.9%)	25.5%	(5.7%-41.2%)	5.3%	(1.3%-9.1%)	2.4%	(-0.1%-4.9%)	7.7%	(2.9%-12.2%)
Larynx*	161	C32	56.7%	(20.0%-76.6%)	16.7%	(-4.8%-33.9%)	73.4%	(-2.1%-93.1%)	0.0%	**- -**	0.0%	**- -**	0.0%	**- -**
Lung*	162	C33-C34	55.8%	(51.2%-60.0%)	13.4%	(10.3%-16.4%)	69.2%	(62.6%-74.7%)	16.4%	(12.0%-20.5%)	3.4%	(1.2%-5.6%)	19.8%	(14.9%-24.4%)
Cervix uteri*	180	C53	**-**	**- -**	**-**	**- -**	**-**	**- -**	8.6%	(0.4%-16.2%)	0.0%	(-3.1%-3.0%)	8.6%	(-0.3%-16.8%)
Kidney, except renal pelvis*	189.0	C64	19.9%	(-11.0%-42.2%)	9.7%	(-9.6%-25.6%)	29.6%	(-21.5%-59.2%)	-2.9%	(-12.3%-5.7%)	1.5%	(-7.4%-9.8%)	-1.4%	(-15.0%-10.7%)
Renal pelvis, ureter, bladder*	189.1, 189.2, 188	C65-C67	56.9%	(39.5%-69.3%)	15.4%	(4.2%-25.4%)	72.3%	(43.1%-86.5%)	5.8%	(-4.0%-14.7%)	0.0%	**- -**	2.9%	(-7.2%-12.1%)
Renal pelvis	189.1	C65	74.6%	(-13.7%-94.3%)	-2.6%	(-25.0%-15.7%)	72.0%	(-87.7%-95.8%)	0.0%	**- -**	0.0%	**- -**	0.0%	**- -**
Ureter	189.2	C66	33.0%	(-59.1%-71.8%)	0.3%	(-46.1%-31.9%)	33.2%	(-141.3%-81.5%)	19.1%	(-20.7%-45.8%)	0.0%	**- -**	16.9%	(-23.9%-44.3%)
Bladder	188	C67	57.4%	(38.4%-70.5%)	21.3%	(8.2%-32.5%)	78.6%	(44.3%-91.8%)	4.5%	(-6.1%-14.1%)	0.0%	**- -**	1.6%	(-9.5%-11.4%)
Myeloid leukemia*	205	C92	14.8%	(-13.7%-36.2%)	20.3%	(1.9%-35.3%)	35.1%	(-12.6%-62.6%)	-0.3%	(-8.9%-7.6%)	-0.1%	(-4.8%-4.4%)	-0.4%	(-10.5%-8.8%)

All cardiovascular diseases	390-459	101-199	18.8%	(15.3%-22.2%)	4.2%	(1.7%-6.6%)	23.0%	(17.5%-28.0%)	6.4%	(5.1%-7.7%)	1.6%	(0.9%-2.4%)	8.0%	(6.5%-9.6%)
All cardiovascular diseases^§^	390-459	101-199	12.4%	(8.9%-15.9%)	2.9%	(0.9%-5.0%)	15.3%	(9.6%-21.0%)	4.5%	(3.3%-5.6%)	1.1%	(0.5%-1.7%)	5.6%	(4.2%-7.0%)
Total tobacco-related cardiovasucular diseases			18.7%	(14.3%-22.8%)	4.5%	(1.5%-7.4%)	23.1%	(16.4%-29.3%)	7.0%	(5.3%-8.7%)	1.8%	(0.8%-2.7%)	8.8%	(6.8%-10.7%)
Ischemic heart diseases*	410-414	120-125	31.8%	(24.9%-37.9%)	12.3%	(7.7%-16.8%)	44.1%	(33.7%-52.8%)	11.6%	(7.9%-15.1%)	3.6%	(1.4%-5.8%)	15.2%	(10.9%-19.3%)
Total stroke*	430-438	160-169	10.6%	(4.8%-16.0%)	-0.1%	(-4.1%-3.8%)	10.4%	(1.4%-18.6%)	5.3%	(3.4%-7.1%)	1.0%	(-0.1%-2.0%)	6.2%	(4.1%-8.4%)
Subarachnoid hemorrhage	430	160	39.5%	(21.3%-53.5%)	3.1%	(-6.3%-11.6%)	42.6%	(15.8%-60.9%)	11.3%	(6.5%-15.9%)	0.1%	(-1.7%-1.9%)	11.5%	(6.2%-16.4%)
Intracerebral hemorrhage	431	161	10.8%	(-1.5%-21.5%)	-2.5%	(-9.8%-4.3%)	8.3%	(-10.2%-23.7%)	6.0%	(2.1%-9.8%)	1.7%	(-0.5%-4.0%)	7.8%	(3.2%-12.2%)
Cerebral infarction	433-434	163	9.5%	(0.6%-17.5%)	0.5%	(-6.1%-6.7%)	9.9%	(-4.5%-22.3%)	3.3%	(0.3%-6.3%)	0.5%	(-1.2%-2.3%)	3.9%	(0.3%-7.3%)
Aortic aneurysm and dissection	441	171	47.4%	(30.3%-60.3%)	18.3%	(6.8%-28.4%)	65.6%	(37.6%-81.1%)	8.2%	(-1.5%-17.0%)	5.4%	(-1.7%-12.0%)	13.6%	(1.4%-24.3%)
Abdominal aortic aneurysm*	441.3, 441.4	171.3, 171.4	52.2%	(18.0%-72.1%)	8.3%	(-11.8%-24.8%)	60.3%	(-1.3%-84.5%)	16.5%	(-9.9%-36.5%)	12.1%	(-8.9%-29.0%)	28.5%	(-5.6%-51.6%)

All respiratory diseases	460-519	J00-J99	13.9%	(8.4%-19.0%)	9.5%	(5.2%-13.7%)	23.4%	(14.5%-31.4%)	4.3%	(1.8%-6.8%)	0.8%	(-0.7%-2.2%)	5.1%	(2.1%-8.0%)
All respiratory diseases^§^	460-519	J00-J99	8.8%	(3.7%-13.9%)	4.9%	(1.0%-8.8%)	13.7%	(4.2%-23.2%)	2.4%	(0.3%-4.5%)	0.8%	(-0.5%-2.1%)	3.2%	(0.6%-5.7%)
Total tobacco-related respiratory diseases			12.4%	(5.8%-18.5%)	6.9%	(1.6%-11.8%)	19.2%	(8.4%-28.7%)	3.6%	(0.5%-6.6%)	1.2%	(-0.7%-3.1%)	4.8%	(1.1%-8.3%)
Pneumonia*	480-486	J12-J18	6.6%	(-0.9%-13.6%)	2.8%	(-3.2%-8.5%)	9.4%	(-3.1%-20.5%)	2.7%	(-0.4%-5.7%)	1.2%	(-0.8%-3.2%)	3.9%	(0.2%-7.5%)
Chronic obstructive pulmonary diseases*	491-492, 496	J41-J44	36.5%	(23.8%-47.1%)	23.8%	(13.5%-32.9%)	60.3%	(39.0%-74.2%)	15.2%	(-1.2%-29.0%)	0.4%	(-5.8%-6.3%)	15.6%	(-2.3%-30.5%)

All digestive diseases	520-579	K00-K93	33.0%	(23.1%-41.6%)	4.1%	(-1.6%-9.4%)	37.1%	(22.6%-48.8%)	7.1%	(3.0%-11.0%)	2.8%	(0.2%-5.3%)	9.9%	(5.0%-14.5%)
All digestive diseases^§^	520-579	K00-K93	4.6%	(-0.5%-9.7%)	0.5%	(-0.7%-1.6%)	5.1%	(-4.0%-14.2%)	0.2%	(-0.7%-1.1%)	0.1%	(-0.5%-0.7%)	0.3%	(-0.9%-1.4%)
Peptic ulcer*	531-533	K25-K27	68.8%	(33.5%-85.3%)	7.4%	(-9.9%-21.9%)	76.0%	(7.5%-93.8%)	2.6%	(-11.9%-15.2%)	1.6%	(-8.3%-10.6%)	4.2%	(-14.1%-19.6%)

*: Tobacco-related diseases selected from the Surgeon General’s Report of 2004 and IARC Monograph volume 83.

§: Population attributable fraction was calculated by summing up attributable fractions estimated for each tobacco-related disease, assuming that the fraction of diseases other than tobacco-related diseases was zero.

### Smoking-attributable Deaths and Diseases in Japan

Of the 1,083,796 total deaths in Japan in 2005 (584,970 males and 498,826 females),^21^ 163,000 (95% CI: 147,000-178,000) male deaths and 33,000 (95% CI: 29,000-38,000) female deaths were estimated to have been caused by smoking, based on the PAF estimates calculated from the relative risk of all-cause mortality. In contrast, summing the disease-specific smoking-attributable deaths yielded smaller estimates; approximately 112,000 (95% CI: 93,000-130,000) male deaths and 19,000 (95% CI: 15,000-21,000) female deaths annually were estimated to have been caused by smoking.

**Figure 3.**
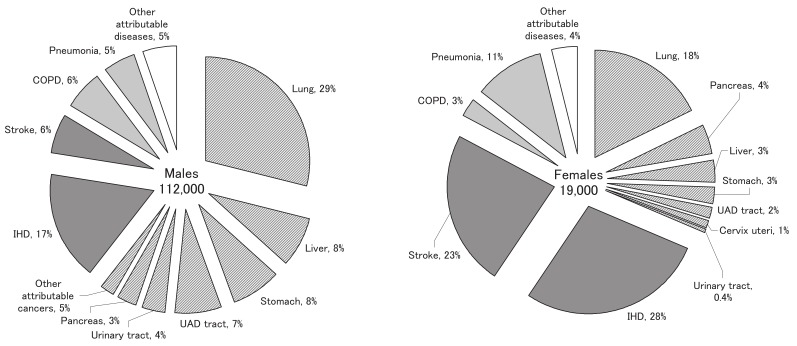
Estimated annual number of smoking-attributable deaths and disease distribution in Japan, for males and females.

Figure 3 shows the disease distribution of the latter set of estimates for smoking-attributable deaths. For males, cancer accounted for approximately 60% of the total smoking-attributable deaths, which was more than double the sum of deaths due to IHD and stroke. Lung cancer accounted for the largest percentage of male smoking-attributable deaths, followed by IHD, liver cancer, stomach cancer, upper aerodigestive (lip, oral cavity, pharynx, or esophagus) cancer, stroke, and COPD. In contrast, for females, IHD and stroke were the leading causes of smoking-attributable deaths, accounting for approximately 50%, whereas cancer accounted for approximately 30%. Lung cancer was the third leading cause, followed by pneumonia, pancreatic cancer, liver cancer, and stomach cancer.

The shaded areas represent cancers, the dark gray areas represent cardiovascular diseases, the light gray areas represent respiratory diseases, and the white areas represent other attributable diseases.

UAD tract: upper aerodigestive tract (lip, oral cavity, pharynx, and esophagus). Urinary tract: renal pelvis, ureter, and bladder. IHD: ischemic heart disease. COPD: chronic obstructive pulmonary disease. Other attributable cancers: cancers of the kidney (except renal pelvis) and larynx, and myeloid leukemia. Other attributable diseases: aortic aneurysm and dissection, and peptic ulcer.

The source of the mortality data was the Vital Statistics of Japan, 2005 (the number of all-cause deaths was 584,970 for males and 498,826 for females).

The number in the center of each chart represents the sex-specific number of smoking-attributable deaths calculated by summing the number of cause-specific deaths weighted by the population attributable fraction of ever-smoking (negative values for the fraction were treated as zero).

The percentage following each disease represents the proportion of smoking-attributable deaths.

## DISCUSSION

The present study analyzed pooled data from three large-scale prospective cohort studies in Japan and estimated the all-cause and disease-specific mortality attributable to smoking. Compared with the results of the historical Hirayama large-scale cohort study,^13^ the estimated age-adjusted relative risks (current smokers vs. never-smokers) in the present study were higher for all-cause mortality [1.6 vs. 1.3 (90% CI: 1.3-1.3) for males, and 1.8 vs. 1.3 (90% CI: 1.3-1.4) for females], for all cancers [2.0 vs. 1.7 (90% CI: 1.6-1.8) for males, and 1.6 vs. 1.3 (90% CI: 1.2-1.4) for females], for IHD [2.2 vs. 1.7 (90% CI: 1.6-1.9) for males, and 3.0 vs. 1.9 (90% CI: 1.7-2.1) for females], for stroke [1.3 vs. 1.1 (90% CI: 1.0-1.1) for males, and 1.8 vs. 1.2 (90% CI: 1.1-1.3) for females]. A possible explanation for the higher relative risks observed in the present study is the increase in exposure levels that has occurred subsequent to the Hirayama study (the baseline survey was carried out in 1965 for the Hirayama study and around 1990 for the present study). The proportion of current smokers who smoked 20 cigarettes per day or more was larger in the present study than in the Hirayama study (71.5% vs. 41.6% for males and 34.3% vs. 8.4% for females, calculated on a person-year basis). Conversely, the proportion who smoked less than 10 cigarettes per day was smaller in the present study than in the Hirayama study (4.7% vs. 10.6% for males and 22.2% vs. 50.6% for females, calculated on a person-year basis). When we compared age at smoking initiation, the proportion of current smokers who started smoking at 19 years of age or earlier was larger in the present study than in the Hirayama study (26.1% vs. 11.9% for males and 7.0% vs. 3.6% for females, calculated based on a person-year basis). Regarding the smoking exposure level for the whole Japanese population, the cigarette consumption per capita among individuals aged 15 years or older increased rapidly from the 1950s to the 1980s,^22^ whereas the smoking prevalence among males decreased during the same period,^23^ suggesting that the number of cigarettes smoked per smoker per day increased during this period. In Japan, the use of filtered cigarettes spread rapidly, and these cigarettes replaced non-filtered cigarettes in the 1960s. The baseline survey for the Hirayama study was carried out in 1965, which was in the middle of this period of change, whereas our baseline survey period (around 1990) occurred long after the completion of the shift to filtered cigarettes. In this sense, the smokers in the present study were considered to have been exposed to less harmful mainstream tobacco smoke than those in the Hirayama study. Previous systematic review reports on the health effects of smoking concluded that there was only a small reduction in lung cancer risk associated with changes in cigarette type,^11^ and only a weak relationship between the cigarette type and coronary heart disease risk.^12^ The HRs of these diseases in the present study were similar to or higher than those in the Hirayama study, and the HRs for the major disease groups, such as all causes, all cancers, and all CVDs, were also higher in the present study. Thus, the shift to filtered cigarettes does not appear to have been influential as far as each of these diseases or the disease groups as a whole are concerned.

One exception regarding the differences between the results of the present study and the Hirayama study is laryngeal cancer. The HR of male current smokers for laryngeal cancer was considerably lower in the present study [5.5 vs. 32.5 (90% CI: 8.7-121.9)]. A possible explanation for this finding is the shift from non-filtered to filtered cigarettes, as mentioned above. Although evidence is lacking, case-control studies conducted in the United States and in several European countries have reported that the use of filters reduced the laryngeal cancer risk by 50%.^24^^-^^26^ One study suggested that the risk reduction produced by filter usage was larger for laryngeal cancer than for lung cancer,^26^ which is consistent with the marked difference between our results and the Hirayama results for laryngeal cancer, but not for lung cancer. An improvement in the prognosis is another possibility. According to a report based on data from a population-based cancer registry in Osaka, Japan, the 5-year relative survival rate for male laryngeal cancer diagnosed in 1975-1977 was 62.1% compared with 80.0% for that diagnosed in 1987-1989.^27^ However, an improvement in survival is common to the cancers of many other sites (e.g., 23.5% to 35.2% for all sites, 22.4% to 38.2% for the pharynx, and 6.0% to 11.7% for the lung).

The prevalence of current smoking in the present study was lower than that reported in the Hirayama study,^13^ [males: 54.4% vs. 74.5% (daily); females: 8.1% vs. 9.7% (daily)]. The prevalence of current smokers in the Hirayama study (carried out in 1965, when there were fewer former smokers) was comparable to the prevalence of ever-smokers in the present study (males: 79.5%; females: 10.5%, from the 1980s to the early 1990s). When we compared the estimate of the PAF of ever-smoking for all-cause mortality in the present study with the Hirayama results (current smoking only), the former was larger both for males (27.8% vs. 17.5%) and for females (6.7% vs. 4.4%). Considering the comparable prevalence of ever-smoking in the present study and current smoking in the Hirayama study, the larger PAFs in the present study would appear to be due to the higher relative risks. Indeed, the relative risks for ever-smokers for all-cause mortality in the present study (vs. never-smokers) were higher than the relative risks for current smokers in the Hirayama study (1.5 vs. 1.3 for males and 1.7 vs. 1.3 for females).

Compared with the annual smoking-attributable mortality in the US from 1997 to 2001,^1^ our estimates of the male disease-specific PAFs of smoking were smaller for cancers of the lip/oral cavity/pharynx (52.0% vs. 74.1%) and the lung (69.2% vs. 87.9%), for pneumonia (9.4% vs. 22.5%), and for COPD (60.3% vs. over 80%), while our estimate was larger for IHD (44.1% vs. 20.8%). Note that the PAFs in the US were calculated from the numbers of deaths, excluding those from passive smoking. When we compared our estimated relative risks with the results of the CPS-II,^28^ upon which the PAF for the US were based, the relative risks for male current smokers (vs. never-smokers) estimated in the present study were lower for all causes [1.6 vs. 2.3 (95% CI: 2.3-2.4)], lung cancer [4.8 vs. 23.2 (95% CI 19.3-27.9)], stroke [1.3 vs. 1.9 (95% CI: 1.6-2.2)], and COPD [3.1 vs. 11.7 (95% CI: 9.1-15.0)]. Given that the prevalence of current smokers among adult males is considerably higher in Japan than in the US (52.8% vs. 25.7%^29^), the smaller PAFs of smoking in the present study were considered to be due to these lower relative risks. In contrast, the relative risks for male current smokers for IHD were similar in the two studies [2.2 vs. 1.9 (1.8: 2.0)]. Thus, the larger male PAF of smoking for IHD recorded in the present study is considered to be due to the higher prevalence of smoking among Japanese males. For females, our estimate of the PAF of smoking was smaller than the US estimates^1^ for many diseases, including lung cancer (19.8% vs. 70.9%), stroke (6.2% vs. 8.7%), and COPD (15.6% vs. over 70%). The relative risks for female current smokers were lower in the present study than in the CPS-II^28^ for all causes [1.8 vs. 1.9 (95% CI: 1.9-2.0)], lung cancer [3.9 vs. 12.8 (95% CI 11.3-14.7)], and COPD [3.6 vs. 12.8 (95% CI 10.4-15.9)], whereas those for stroke were similar [1.8 vs. 1.8 (95% CI: 1.6-2.1)]. The prevalence of female current smokers is considerably lower in Japan than in the US (13.4% vs. 21.5%^29^). Thus, the lower PAFs of smoking in Japanese females for lung cancer and COPD are considered to be due to both the lower relative risks and the lower prevalence of smokers. In the case of stroke, the lower PAF was thought to be due to the lower smoking prevalence.

The lower relative risks associated with smoking for Japanese populations compared with those for Western populations have been well documented by previous studies for all causes,^6^^,^^30^ total cancers,^7^ and lung cancer.^15^^,^^31^^,^^32^ A commonly proposed reason for this finding is the lower exposure level among Japanese smokers.^15^^,^^32^ However, the difference in relative risks is reported to remain even after adjustment for duration of smoking and daily cigarette consumption,^15^ or stratification by dose of exposure.^30^^,^^31^ Other proposed reasons include the possibility of a higher level of passive smoking in Japan (i.e., a higher risk for non-smokers), the misclassification of former smokers as never-smokers (causing an apparent increase in the risk to non-smokers) and a lower genetic susceptibility to tobacco smoke among the Japanese. It is also possible that COPD tends to be underreported as a cause of death on death certificates.

There are several limitations to the present study that could have been potential sources of uncertainty in the estimation of the fraction and the number of smoking-attributable deaths. First, the smoking prevalence used for the estimation of the PAFs was obtained from our cohort data, the baseline survey for which was conducted from the 1980s to the early 1990s. The reason for using cohort data was the need to obtain the prevalence among those who died of a given cause of death.^20^ There have been recent changes in the prevalence of smoking in Japan, and a decreasing trend for males is becoming evident. Although the pooled smoking prevalence in the present study was comparable to the national representative adult prevalence around the year 1990 (e.g., 53.1% for males and 9.7% for females in 1990),^33^ recent corresponding values were lower for males and higher for females (43.3% for males and 12.0% for females in 2004).^34^ On the basis of the national representative smoking prevalence data in 2004 and the relative risks for all-cause mortality in the present study, the PAF of ever-smoking was 25.2% for males and 11.0% for females. The corresponding value based on the prevalence data in the present study (i.e., the prevalence among all participants, not among those who died) was 29.1% for males and 7.2% for females. Thus, the PAFs of smoking in recent calendar years for the Japanese population are probably smaller for males and larger for females, as compared with our estimates.

The information on the smoking status of our participants was collected only at the baseline. Smoking cessation or initiation during the follow-up period might have led to an underestimation of the relative risks of current or former smokers and, conversely, smoking re-initiation during the follow-up period might have caused an overestimation of the relative risk of former smokers. A Japanese cohort study that examined smoking status 5 years after the baseline survey demonstrated that the shift from current to former smokers was considerably more frequent than either the shift from never-smokers to current smokers or the shift from former to current smokers.^35^ This suggests the possibility of underestimating the relative risks of current smokers. However, our relative risk estimate of male current smokers for lung cancer was similar to that obtained by pooling the data from Japanese case-control studies,^36^ which implies that the possible change in smoking status had only a limited influence, at least on the lung cancer relative risks. It remains possible that the relative risks of current smokers were underestimated for diseases with a risk that decreases more rapidly after smoking cessation compared to lung cancer.

We excluded participants with unknown smoking status (5% of males and 12% of females). In our preliminary analysis, we calculated the lung cancer mortality rate among participants with unknown smoking status; the value was found to be similar to the mortality rate among current smokers for males, whereas for females it was between the mortality rates of former smokers and never-smokers. If the other risk factors of lung cancer were evenly distributed, it can be assumed that most of the males with unknown smoking status were actually smokers, whereas the females with unknown smoking status were not strongly biased toward smokers or never-smokers. Thus, the prevalence of male smokers could have been underestimated by the selective exclusion of smokers. However, the extent of this effect was considered to be small since the proportion of male participants with unknown smoking status was correspondingly small.

Since the relative risks estimated in the present study were adjusted only for age, other potential confounding factors might have influenced our results. One such possible confounding factor was cohort, although this might have been negligible because the HRs adjusted for age and cohort did not differ from those adjusted only for age [e.g., the age- and cohort-adjusted HR of lung cancer for current smokers was 4.8 (95% CI: 3.9-5.9) for males and 3.8 (95% CI: 3.0-4.9) for females]. Our relative risk and PAF estimates for a specific disease might have been overestimated if its risk factors were positively correlated with smoking (i.e., alcohol consumption for esophageal cancer). For several disease groups, the age-adjusted relative risks of current smokers (vs. never-smokers) have been reported to be slightly higher than the multivariate adjusted values (i.e., all causes,^6^^,^^30^ stomach cancer,^37^ and stroke^10^^,^^38^), suggesting the existence of risk factors associated with smoking. In contrast, it is possible that the list of tobacco-related diseases might overlook non-established smoking-attributable diseases or disease sub-categories. Thus, our PAF estimates of all-cause mortality calculated using the relative risk of all-cause mortality itself [i.e., equation (1) in the Methods section] might have included overestimates, whereas the PAF calculated by summing the disease-specific PAFs [i.e., equation (2)] might have included underestimates.

For diseases with a relatively long duration (i.e., a time lag from incidence to death), high HRs in former smokers could be due to the “ill-quitter” effect; that is, those individuals who developed these diseases might have quit smoking because of the illness. We analyzed our data excluding deaths within 5 years of follow-up and confirmed that there was no major change in the relative risks of former smokers.

The sample sizes were small for relatively rare diseases, particularly among females. We either could not estimate HRs, or the estimated HRs had a wide CI, for female mortality from cancers in the lip/oral cavity/pharynx, esophagus, larynx, and kidney (except renal pelvis), myeloid leukemia, abdominal aortic aneurysm, COPD, and peptic ulcer. However, since these causes of death accounted for a small proportion of the total number of deaths observed in the present study (2% of the total female deaths), we consider the instability of the HRs to have had only a weak influence on our estimates of the disease distribution of smoking-attributable deaths.

Regarding the generalizability of our PAF estimates, some of the participants in the present study were recruited not from the general population but rather from those undergoing health check-ups (Table 1). Health check-up examinees might have different relative risks to those of the general population to which they belong. For example, a previous study using the JPHC cohort examined the differences in relative risks between health check-up examinees and the entire cohort, and revealed that the relative risk of all-cause mortality for current smokers (vs. never-smokers) was 24% higher for health check-up examinees.^39^ These types of difference might have influenced our relative risk estimates.

Another issue regarding generalizability is age. The age distribution of participants in the present study was slightly different to that of the Japanese population as a whole. Compared with the Japanese population aged 40-79 years in 1983-1994, the proportion of those aged 70-79 years was smaller among the participants in the present study (7.5% vs. 10.9% for males and 8.0% vs. 14.4% for females). Generally, the prevalence of current smokers was lower among the group aged 70-79 years than among the younger age groups. We used the age-pooled smoking prevalence to calculate the PAFs, which might have led to the inclusion of slight overestimations.

The reason for the small proportion of individuals aged 70-79 years among the participants in the present study was that this age group was only covered by the 3-pref and JACC cohorts. We analyzed the differences between the groups of cohorts with and without this age group (3-pref + JACC vs. JPHC-I + JPHC-II) in terms of the age-adjusted HR of the current smokers (vs. never-smokers) for all-cause mortality, limiting to the common baseline age groups (40-59 years old). The calculated HRs were similar [males: 1.8 (95% CI: 1.6-2.0) for 3-pref + JACC, 1.8 (95% CI: 1.6-2.0) for JPHC-I + JPHC-II; females: 1.9 (95% CI: 1.6-2.2) for 3-pref + JACC, 1.8 (95% CI: 1.5-2.1) for JPHC-I + JPHC-II]. The prevalence of current smokers in the two groups of cohorts was not widely different (males: 58.8% for 3-pref + JACC, 54.5% for JPHC-I + JPHC-II; females: 8.8% for 3-pref + JACC, 8.2% for JPHC-I + JPHC-II). Therefore, the influence of using partial data for the group aged 70-79 years was considered to be small.

The generalizability of our PAF estimates to the age groups that were not covered by the present study (i.e., those under 40 or over 79 years old) is limited. We estimated the number of deaths attributable to smoking using the all-age number of deaths in Japan. In this calculation, the influence of the group aged under 40 years was negligible because it accounted for only a small part of the all-age mortality in Japan (2.6% in 2005). The group aged over 79 years was partly covered by the present study in terms of attained age since the follow-up period was on average 10 years. According to a previous study that used the same dataset employed in the present study, the all-cause mortality rate ratios of current smokers vs. never-smokers were similar for the groups aged 40-69 years and 70 years or older (calculated using the attained age).^40^ The smoking prevalence among those aged 70-79 years in the present study was not notably different to the national data for those aged 70 years or older (42.5% vs. 38.8% for males and 8.5% vs. 7.2% for females).^33^ Thus, we believe that approximating the number of smoking-attributable deaths for all ages based on our PAF estimates is a valid approach.

In conclusion, we used the pooled data from three large-scale cohort studies in Japan to demonstrate that the estimated smoking-attributable fraction of all-cause mortality among individuals aged 40-79 years was 27.8% for males and 6.7% for females. The corresponding values calculated by summing the disease-specific smoking-attributable fractions were 19.1% for males and 3.6% for females. These results confirmed that the health burden of smoking is still large among Japanese males. Considering the high prevalence of male current smokers and the increasing prevalence of young female current smokers, effective tobacco controls and quantitative assessments of the health burden of smoking should be continuously implemented in Japan.
